# Bovine milk derived skimmed milk powder and whey protein concentrate modulates *Citrobacter rodentium* shedding in the mouse intestinal tract

**DOI:** 10.7717/peerj.5359

**Published:** 2018-07-27

**Authors:** Julie Cakebread, Alison Hodgkinson, Olivia Wallace, Megan Callaghan, Daralyn Hurford, Robert Wieliczko, Paul Harris, Brendan Haigh

**Affiliations:** 1 Dairy Foods Team, Food & Bio-based Products, AgResearch, Hamilton, New Zealand; 2 Miraka Limited, Taupo, New Zealand

**Keywords:** WPC, SMP, Bioactives, *C. rodentium*, Mouse

## Abstract

Skimmed milk powder (SMP) and whey protein concentrate (WPC) were manufactured from fresh milk collected from cows producing high or low Immunoglobulin (Ig) A levels in their milk. In addition commercial products were purchased for use as diluent or control treatments. A murine enteric disease model (*Citrobacter rodentium*) was used to assess whether delivery of selected bioactive molecules (IgA, IgG, Lactoferrin (Lf)) or formulation delivery matrix (SMP, WPC) affected faecal shedding of bacteria in *C. rodentium* infected mice. In trial one, faecal pellets collected from mice fed SMP containing IgA (0.007–0.35 mg/mL), IgG (0.28–0.58 mg/mL) and Lf (0.03–0.1 mg/mL) contained fewer *C. rodentium* (cfu) compared to control mice fed water (day 8, *p* < 0.04, analysis of variance (ANOVA) followed by Fisher’s unprotected least significant difference (ULSD)). In trial two, WPC containing IgA (0.35–1.66 mg/mL), IgG (0.58–2.36 mg/mL) and Lf (0.02–0.45 mg/mL) did not affect *C. rodentium* shedding, but SMP again reduced faecal *C. rodentium* levels (day 12, *p* < 0.04, ANOVA followed by Fisher’s ULSD). No *C. rodentium* was detected in sham phosphate-buffered saline inoculated mice. Mice fed a commercial WPC shed significantly greater numbers of *C. rodentium* over 4 consecutive days (Fishers ULSD test), compared to control mice fed water. These data indicate that SMP, but not WPC, modulates faecal shedding in *C. rodentium-*infected mice and may impact progression of *C. rodentium* infection independently of selected bioactive concentration. This suggests that food matrix can impact biological effects of foods.

## Introduction

Milk is a complex food, containing not just a supply of proteins, lipids and carbohydrates to support growth of the suckling young, but also a range of substances that contribute to digestive and immune function (Tunick & Van Hekken, 2015; Wheeler et al., 2007). These include bioactive molecules such as immunoglobulins (Igs) (Farrell et al., 2004; Telemo & Hanson, 1996) and lactoferrin (Lf) (Giansanti et al., 2016) which play an important role in gastrointestinal (GI) maturation and protection against microbial pathogens. Other immune-related proteins including defensins, cathelicidins, calgranulins, lipopolysaccharide-binding protein and the RNases also have important roles in mucosal defence (Wheeler et al., 2012). For this study we have chosen IgA, IgG and Lf as relevant ‘markers’ of bioactivity (bioactive) in our milk products.

In human milk the most prevalent Ig is IgA, which is secreted as a dimer complexed with secretory component (Brandtzaeg, 2013; Cakebread, Humphrey & Hodgkinson, 2015). Secretory IgA in human milk protects the infant against enteric infection (Noguera-Obenza & Cleary, 2001) and is thought to act within the intestinal lumen to maintain microbial homeostasis (Mestecky, 2001; Noguera-Obenza & Cleary, 2001). Cows’ milk differs in composition from human milk, containing relatively low concentrations of IgA and higher concentrations of IgG which is important for passive immunisation of the new born calf (Korhonen, Marnila & Gill, 2000). Lf, an iron-binding protein, is found in both human and bovine milk with reported roles including GI development, antimicrobial activity and immunomodulation (Giansanti et al., 2016; Lonnerdale & Iyer, 1995). Like IgA, Lf is more prevalent in human milk than bovine milk, although, levels vary between individuals and stage of lactation (Broadhurst et al., 2015; Cheng et al., 2008).

Humans are unique in the widespread practise of consuming the milk of other species. The implications of this for the health and well-being of humans are still not fully understood. The Igs in cows’ milk have been associated with human health benefits (Korhonen, Marnila & Gill, 2000), however, most studies have focused on the benefits of colostrum and IgG (Rathe et al., 2014). IgA from bovine milk also has the potential to confer a health benefit to human consumers, in part, due to the secretory IgA molecule being more stable to degradation by digestive enzymes than IgG (Lindh, 1975). This property aids retention of IgA which remains active within the digestive tract after ingestion. However, while the potential health benefits of IgA, IgG and Lf in bovine milk have been proposed (Cakebread, Humphrey & Hodgkinson, 2015), they have yet to be demonstrated in humans studies.

The concentration of bioactive molecules in milk varies substantially among cows (Berry et al., 2013; Ploegaert et al., 2010; Wijga et al., 2013) and this is influenced by both genetic and environmental factors. For example, some cows consistently produce milk with naturally higher IgA levels, even approaching the concentration found in human milk. However, when milk is processed into products, such as skimmed milk powder (SMP) or whey protein concentrate (WPC), standard processing methods have been shown to alter milk proteins and potentially associated bioactivity (Davis & Williams, 1998; Sousa, Delgadillo & Saraiva, 2014).

We hypothesised that both the levels of selected bioactive molecules and the associated matrix would impact the biological function of the material. In a murine model of enteric disease elicited by *Citrobacter rodentium*, mice were fed SMP or WPC products containing a range of IgA, IgG and Lf concentrations. Potential impact on infection progression was measured by faecal shedding of *C. rodentium*.

## Materials and Methods

### Collection of milk for preparation of SMP and WPC

Milk for in-house SMP and WPC products was obtained from Jersey and Friesian cows grazed on pasture and located on one farm in the central North Island region of New Zealand, as part of a commercial milking herd. The cows were at mid lactation and milked on a twice daily regime. Individual cows were screened for IgA levels, as a representative marker of selected bioactive protein content. For preparation of the SMP, milk was collected on two occasions from three cows with high levels of IgA in their milk (SMP high) and three cows with low levels of IgA (SMP Low). Milk was pooled from each group and stored at 2–4 °C until processed. For processing of milk into WPC a larger volume was required. Milk was collected on two occasions, on two consecutive days, from 18 cows producing high levels of IgA (WPC high) in their milk. This group included the cows milked for the SMP product. Milk was pooled and stored at 2–4 °C until processed.

### Milk processing and reconstitution

#### Preparation of high and low SMPs

Milk pools were pasteurised using a tubular heat exchanger pasteuriser unit (Food Pilot, Palmerston North, New Zealand) then skimmed with a disk-stack centrifuge and evaporated to between 40% and 45% solids, at a temperature of 55 °C. The concentrated milk preparations were spray-dried using a GEA Minor spray-drier at a pre-heat temperature of 50 °C, and an oven inlet temperature of 180 °C. The high and low SMP products were stored in sealed light proof bags at ambient temperature.

#### Preparation of high WPC

The milk pool was pasteurised and skimmed, as described above. Batches of approximately 180 L were transferred to a heated, stirred process vessel, and maintained at a temperature of 40 °C. Dilute hydrochloric acid was added to the milk with gentle stirring until a pH value between 4.6 and 4.8 was obtained. The milk was left for 1 h with occasional stirring, and the pH re-checked and adjusted if necessary. Whey was decanted off the casein curd and filtered through a coarse sieve to remove any large pieces of curd. Whey was then clarified with a disk-stacked centrifuge to remove any fine casein curds before ultra-filtering with a 10 kDa membrane (Koch membrane systems, Wilmington, MA, USA) to a brix of approximately 25% and then diafiltered against six volumes of reverse osmosis water. The final concentrate was adjusted with Sodium hydroxide (NaOH) to a pH of 6.6 and stored either chilled or frozen until spray-drying, as described above for SMP products. The WPC product was stored in sealed light proof bags at ambient temperature.

#### Preparation of Low WPC

Commercial WPC was reconstituted in 40 °C water to obtain a protein level, of 4.55%, determined by Milkoscan (FT1, Foss Analytical, NZ), and stirred until all powder was thoroughly dissolved. The solution was then heat-treated by pumping through a series of stainless steel tubing immersed in temperature-controlled water baths set to obtain a temperature of 80 °C for 15 s. After heating, the protein level was adjusted to 3.27% by adding pasteurised water and stored in sealed light proof bags at ambient temperature.

#### Preparation of SMP and WPC for trials

To obtain SMPs with a range of selected bioactive levels, High SMP was blended with Low SMP (prepared as described earlier) at different ratios: 100:0—High SMP, 65:35—mid-high SMP, 35:65—mid-low SMP, 10:90—Low SMP. Similarly, High WPC and Low WPC were blended (prepared as described earlier) to produce WPCs with a range of selected bioactive levels; 100:0—High WPC, 60:40—mid-high WPC, 25:75—mid-low WPC, 0:100—Low WPC (Table 1). In addition, we purchased commercial SMP and colostrum for comparison in our trials. Commercial WPC (not heat treated) was also included as a treatment. All products and blends were reconstituted using molecular biology-grade (milli Q) water. Fat, protein and lactose levels were measured using the Milkoscan (FT1; Foss Analytical, Cambridge, New Zealand). SMP was reconstituted at 10% w/v total solids and WPC was reconstituted at 3.7 % w/v total solids. Therefore, all treatments (except colostrum) prepared for the trials were fed to mice using the same protein concentration (3.2%). The dose for colostrum was determined according to the manufacturer’s recommendations for daily intake. So, for a young girl (average weight 14 kg) a recommended intake is three g per day or 0.2 g/kg body weight. This was scaled down for the mouse (average weight 21 g, average daily liquid intake 3.1 mL) to give an equivalent dose; calculated to be 4.2 mg colostrum per mouse per day.

**Table 1:** Composition of SMP, WPC and commercial products after reconstitution and blending.

	Blend	Bioactive content (mg/mL)		
SMP	High SMP:low SMP (%)	IgA	IgG	Lf
High SMP*	100:0	0.35	0.58	0.1
Mid-high SMP	65:35	0.23	0.41	0.07
Mid-low SMP	35:65	0.13	0.35	0.05
Low SMP	10:90	0.07	0.28	0.03
Colostrum	–	nd	0.07	nd
Commercial SMP	–	nd	nd	nd

WPC	High WPC:low WPC (%)			
High WPC	100:0	1.66	2.36	0.45
Mid-high WPC	60:40	1.08	1.66	0.29
Mid-low WPC	25:75	0.53	1.07	0.12
Low WPC	0:100	0.22	0.75	0.02
High SMP*	100:0	0.35	0.58	0.1
Commercial WPC		0.43	1.10	0.08

**Notes:**

SMP and WPC preparations were blended to produce treatments with a range of three selected bioactive levels (IgA, IgG and LF). Milk products were normalised to 3.2% protein.

* Indicates the same product used in both trials; nd—non detected.

Levels of IgA, IgG and Lf in the reconstituted protein-normalised liquids were determined by enzyme linked immunosorbent assay (ELISA) using commercially supplied kits (Bethyl Laboratories, Montgomery, TX, USA), as per the manufacturer’s recommendations (Table 1).

### Preparation and administration of *C. rodentium*

A nalidixic acid-resistant strain of *C. rodentium* (DBS100) was obtained from P. Fineran, University of Otago, and was grown overnight in Luria-Bertani (LB; Fort Richard Laboratories, Auckland, New Zealand) broth at 37 °C, then transferred into LB broth containing 50 μg/mL nalidixic acid (Sigma-Aldrich, Auckland, New Zealand), and streaked onto an LB-agar plate (Fort Richard Laboratories, Auckland, New Zealand) containing 50 μg/mL nalidixic acid. Single nalidixic acid-resistant colonies were serially selected a further two times (Wiles et al., 2004). A selected single circular colony of *C. rodentium* was then grown overnight in LB broth, pelleted by centrifugation at 500 *g* for 15 min, and resuspended in phosphate-buffered saline (PBS; pH 7.2, Oxoid; ThermoFisher Scientific, Auckland, New Zealand). Mice were orally inoculated using a gavage needle containing 150 μL of *C. rodentium* in PBS (2 × 10^9^ bacteria/inoculum) (Bouladoux, Harrison & Belkaid, 2017) which was freshly prepared for each oral gavage. The stock suspension was diluted and plated onto nalidixic acid-containing LB agar, incubated overnight at 37 °C and the viable count enumerated to confirm the number of viable bacteria inoculated.

### Mouse feeding trials

All animal manipulations and procedures were approved by the Ruakura Animal Ethics Committee (RAEC #13644, #13701). Female BALB/c mice were bred within the Ruakura Small Animal facility at AgResearch in Hamilton, New Zealand. All mice used in these studies were housed under quarantine and in micro-isolator cages throughout the experimental period. Mouse chow was available ad libitum.

Two trials were conducted: (1) the SMP trial and (2) the WPC trial. For each trial mice (aged 8–10 weeks) were randomised into seven treatment groups of 16 mice and a control (no infection) group of eight mice. Animals were housed in cages of up to four animals per cage.

The daily liquid intake volumes were recorded and an average per/mouse estimated (intake per group divided by number of mice per cage Table 2). Fluid intake was measured by weighing the bottles each day to determine the volume of liquid remaining. After weighing, the bottles were replaced with fresh liquid. The control groups were given water to drink. The treatment animals had WPC or SMP as their only source of liquid. The bottles containing WPC and SMP were replaced daily with freshly mixed powder/water. To ensure consistency, sufficient powder was pre-weighed and aliquoted prior to the experiment, for the duration of the trial. The correct amount of water was added for mixing each day. Treatment groups were fed their designated liquid for 10 days before inoculation of *C. rodentium* by gavage, and then until trial end. Animals were denied food overnight prior to gavage but their milk treatments remained. On inoculation day all treatment groups were inoculated with *C. rodentium* in PBS (pH 7.2), and the control (no infection) group was inoculated with PBS only.

**Table 2:** Average daily liquid intake per mouse and calculated IgA, IgG and LF intake.

Treatment	Average liquid intake (mL/mouse/day)	Intake (μg/gram mouse/day)		
SMP		IgA	IgG	Lf
Water	2.96	0	0	0
SMP high*	4.09	79	132	23
SMP mid-high	3.74	47	84	14
SMP mid-low	3.68	26	71	10
SMP low	3.71	14	57	6
Colostrum commercial	3.78	0	15	0
SMP commercial	3.50	0	0	0
Water no infection	2.85	0	0	0
**WPC**				
Water	3.18	0	0	0
WPC high	3.66	309	440	84
WPC mid-high	3.81	214	328	57
WPC mid-low	3.97	109	219	25
WPC low	4.26	48	163	4
SMP high*	4.01	65	107	18
WPC commercial	3.68	88	224	16
Water no infection	2.96	0	0	0

**Notes:**

SMP and WPC preparations were blended to produce treatments with a range of IgA, IgG and LF levels. Average amount per mouse was calculated from intake volume per cage.

SMP, skimmed milk powder; WPC, whey protein concentrate; Ig, immunoglobulin.

* Indicates the same product used in both trials.

The mice were weighed at the trial start, then seven days before bacterial inoculation, immediately before inoculation, daily for the first four days after inoculation, and then every second day thereafter, until end of trial. The behaviour and stance of each mouse was monitored daily throughout the trial for signs of discomfort using a health score (activity; posture; coat; breathing).

### Analysis of faecal shedding

*Citrobacter rodentium* colonization was assessed by enumerating viable bacteria from faecal samples, based on the assumption that shedding of *C. rodentium* in the faeces parallels colonization (Mundy et al., 2005). Faecal pellets were collected at the start of the trial, immediately before inoculation, and then every second day after inoculation until trial end (10 days following inoculation for SMP trial and 14 days for WPC trial). Pellets were collected by placing each mouse in a separate aerated container until they had passed two to three faecal pellets. Faecal pellets were weighed and then homogenised in PBS to achieve a suspension of 100 mg pellet per mL. A series of dilutions of the suspension were plated onto LB-agar containing nalidixic acid. The colonies (viable count) present after incubation at 37 °C for 24 h were enumerated and reported as cfu/g faeces.

### Chemicals and reagents

Hydrochloric acid (CAS 7647-01-0), NaOH (CAS 1310-73-2) and Nalidixic acid (CAS 389-08-2) were obtained from Sigma-Aldrich (Auckland, New Zealand).

ELISA kits were obtained from Bethyl Laboratories (Montgomery, TX, USA), and PBS tablets from Oxoid; ThermoFisher Scientific (Auckland, New Zealand).

### Statistical design

Comparisons between treatment groups were performed using analysis of variance (ANOVA) using GenStat (Genstat for Windows 17th Edition; VSN International, Hemel Hempstead, UK). Post hoc analysis used Fisher’s unprotected least significant difference (ULSD). Error bars illustrate standard errors of differences of means (sed).

## Results

### Characterisation of SMP and WPC products

Following pilot scale processing, all reconstituted blended SMPs contained 3.2% protein, 4.4% lactose, but had no detectable fat. Levels of IgA, IgG and Lf in blended liquids and the commercially sourced colostrum and SMP products are listed in Table 1. Blended SMPs contained IgA ranging from 0.07 to 0.35 mg/mL, IgG ranging from 0.28 to 0.58 mg/mL and Lf ranging from 0.03 to 0.1 mg/mL. Commercial SMP had undetectable levels of IgA, IgG and Lf, while commercial colostrum only had detectable IgG (0.07 mg/mL).

Reconstituted WPC contained less than 0.2% fat, and lactose was not detectable. Levels of IgA, IgG and Lf in blended liquids and the commercial WPC are listed in Table 1. Blended WPCs contained IgA ranging from 0.35 to 1.66 mg/mL, IgG ranging from 0.58 to 2.36 mg/mL and Lf ranging from 0.02 to 0.45 mg/mL. When reconstituted to 3.2% protein, the commercial WPC contained 0.43 mg/mL IgA, 1.10 mg/mL IgG and 0.08 mg/mL Lf.

For the animal trials, the maximum estimated daily dose of IgA, IgG and Lf was more than four times higher in the WPC treatments compared with the SMP treatments (Table 2). Animals fed commercial WPC received IgA, IgG and Lf concentrations that were intermediate between the mid-low and mid-high WPC treatment groups.

### Mouse weights and liquid intake during the trial

Skimmed milk powder feeding commenced 10 days prior to inoculation with *C. rodentium* (oral gavage) and continued for 10 days after, which was the expected time of peak shedding as determined by a pilot trial (Fig. S1). Towards the end of the trial it was apparent that peak shedding differed slightly from the pilot trial. The WPC trial was performed following the SMP trial and the time was extended to provide more extensive data on the period following peak *C. rodentium* shedding.

Weight measurements (Figs. S3 and S4) and liquid intake (Table 2) indicated the infection did not adversely affect the health or growth of the mice over the course of the experiment. Mice were weighed every day for 4 days and then every second day to monitor infection-induced weight loss due to poor appetite and/or dehydration. There was no significant differences between start weight of groups in the SMP trial or groups in the WPC trial. The average weights of water-fed infected mice was significantly lower than water fed no-infection controls on days 2 (*p* = 0.02) and day 3 (*p* = 0.02) for SMP trial and on day 2 of the WPC trial (*p* = 0.03) (Figs. S3 and S4). Any weight loss was transient and animals’ weight quickly returned to normal. The non-infected mice that were given water steadily gained weight (Figs. S3 and S4). Overall, animals drinking SMP and WPC consumed a higher volume of liquid compared with those mice drinking water, which may suggest increased palatability of milk diets (Table 2). The intake of water for the no infection control groups were similar between the WPC and SMP trials (*p* = 0.3) but water intake in groups infected with *C. rodentium* was different (average water + infection for SMP trial and WPC trial was 2.9 mL/mouse and 3.2 mL/mouse, respectively; *p* = 0.03, Student’s t-test, equal variance).

### Effect of SMP on *C. rodentium* shedding over time

**Figure 1:**
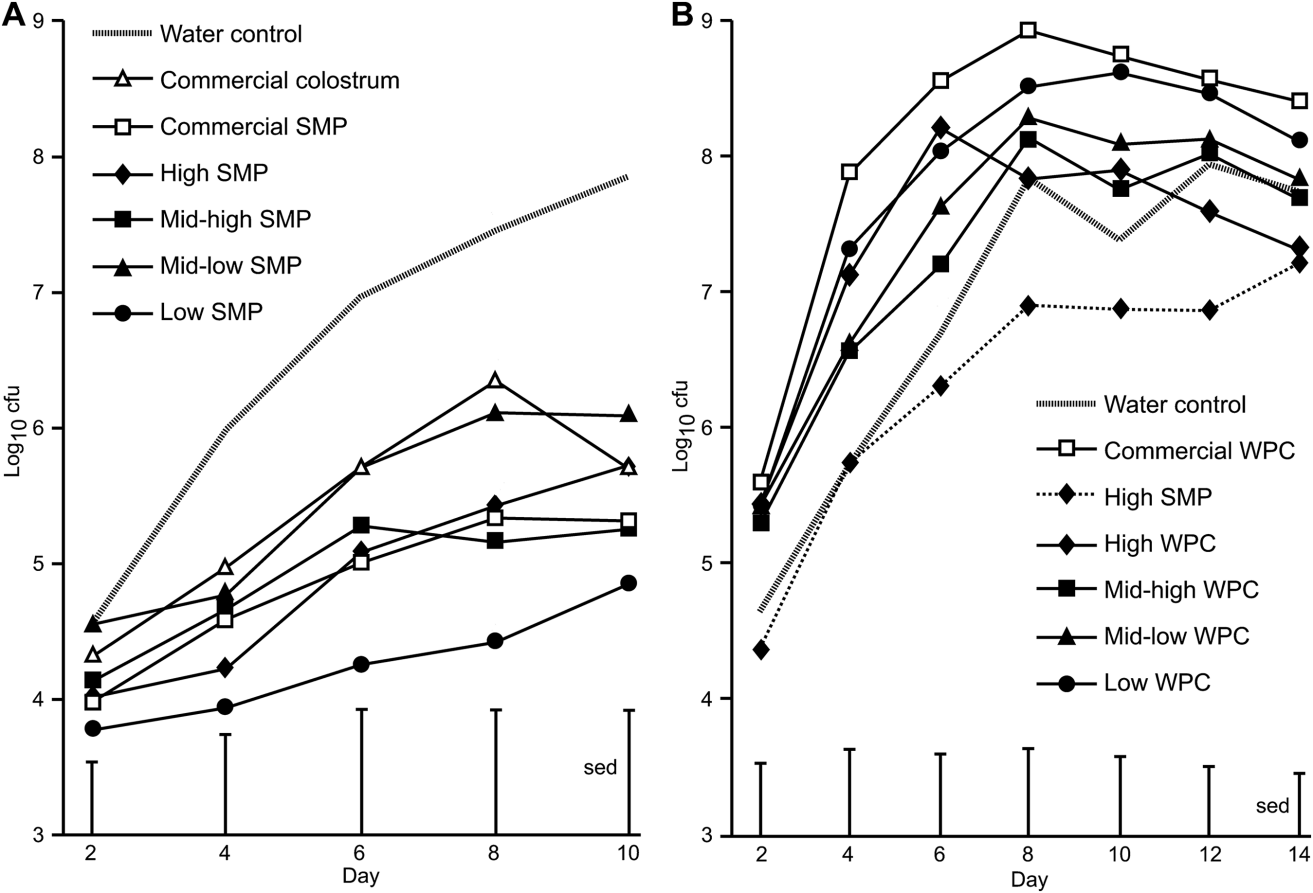
Comparison of Citrobacter rodentium infection between mice supplemented with SMP or WPC. *C. rodentium* was enumerated from faecal collections (cfu/g faeces). (A) Colonisation in mice supplemented with treatments of SMP over 10 days. Grey dash—water control, filled diamond—SMP high, filled square—SMP mid-high, filled triangle—mid-low SMP, filled circle—Low SMP, empty triangle—Commercial colostrum, empty square—Commercial SMP. *n* = 16 per group. (B) Colonisation in mice supplemented with treatments of WPC over 14 days. Grey dash—water control, filled diamond and line—High WPC, filled square—mid-high WPC, filled triangle—mid-low WPC, filled circle—Low WPC, filled diamond and dots—High SMP, empty square—Commercial WPC. *n* = 16 per group.

On Day 0, all groups received a bacterial inoculation or sham PBS inoculation via oral gavage. No *C. rodentium* was detected in sham PBS inoculated mice. The average viable count of *C. rodentium* present in the faecal pellets of the mice for each of the treatment groups over the course of the infection period are presented in Fig. 1A. For SMP fed mice, differences were observed between water controls and the high SMP, mid-high SMP, low SMP and commercial SMP treatment groups on day 8 and water controls and low SMP treatment group on day 10 (Fig. 1A). The mean *C. rodentium* viable count for the each of the SMP groups (except for the mid-low treatment) was significantly lower than water-fed infected controls (ANOVA followed by Fisher’s ULSD). We could not detect significant differences in bacterial viable counts with regard to levels of IgA, IgG or Lf in the SMP, or colostrum treatment.

### Effect of WPC on *C. rodentium* shedding over time

In the WPC trial, animals in the WPC treatment groups excreted equal or increased levels of faecal *C. rodentium* compared to water-fed infected controls. This trial was extended to observe progression of infection. Significant differences were observed between high SMP and commercial WPC (day 4–day 12, *p* < 0.02 ANOVA followed by Fisher’s ULSD), Fig. 1B and Table S3). No *C. rodentium* was detected in sham PBS inoculated mice.

### Evaluating the effect of milk matrix on *C. rodentium* shedding over time

The second trial also enabled us to compare treatments that delivered similar amounts of selected bioactive but in a different product matrix (SMP versus WPC). For example, Table 1 shows IgA content of high SMP to be 0.35 mg/mL which sits between mid-low and low WPC which contain 0.53–0.22 mg/mL IgA respectively. Mid-high SMP contains 0.23 mg/mL IgA, and low WPC 0.22 mg/mL IgA. Table 2 shows estimated selected bioactive IgA intake for SMP mid-high fed mice to be 47 μg/g/mouse/day which is comparable to WPC low, at 48 μg/g/mouse/day.

The WPC trial included a High SMP treatment group where the consumption of IgA, IgG and Lf (65, 107 and 18 μg/gram mouse/day respectively) was intermediate between the mid-low WPC (109, 219 and 25 μg/gram mouse/day respectively) and Low WPC (48, 163 and 4 μg/gram mouse/day) treatment groups. Lower *C. rodentium* counts (cfu) (as determined by viable counts) were observed from animals receiving High SMP, but not for those receiving the mid-Low and Low WPC treatments. In addition, the High SMP group had significantly less *C. rodentium* counts (cfu) compared to the commercial WPC fed animals (Fig. 1B), which were the treatment groups containing the most similar selected bioactive content (IgA, IgG and Lf at 88 μg, 224 μg and 16 μg/gram mouse/day respectively).

### Effect of SMP or WPC on magnitude (maximum viable count) of *C. rodentium* faecal shedding

**Figure 2:**
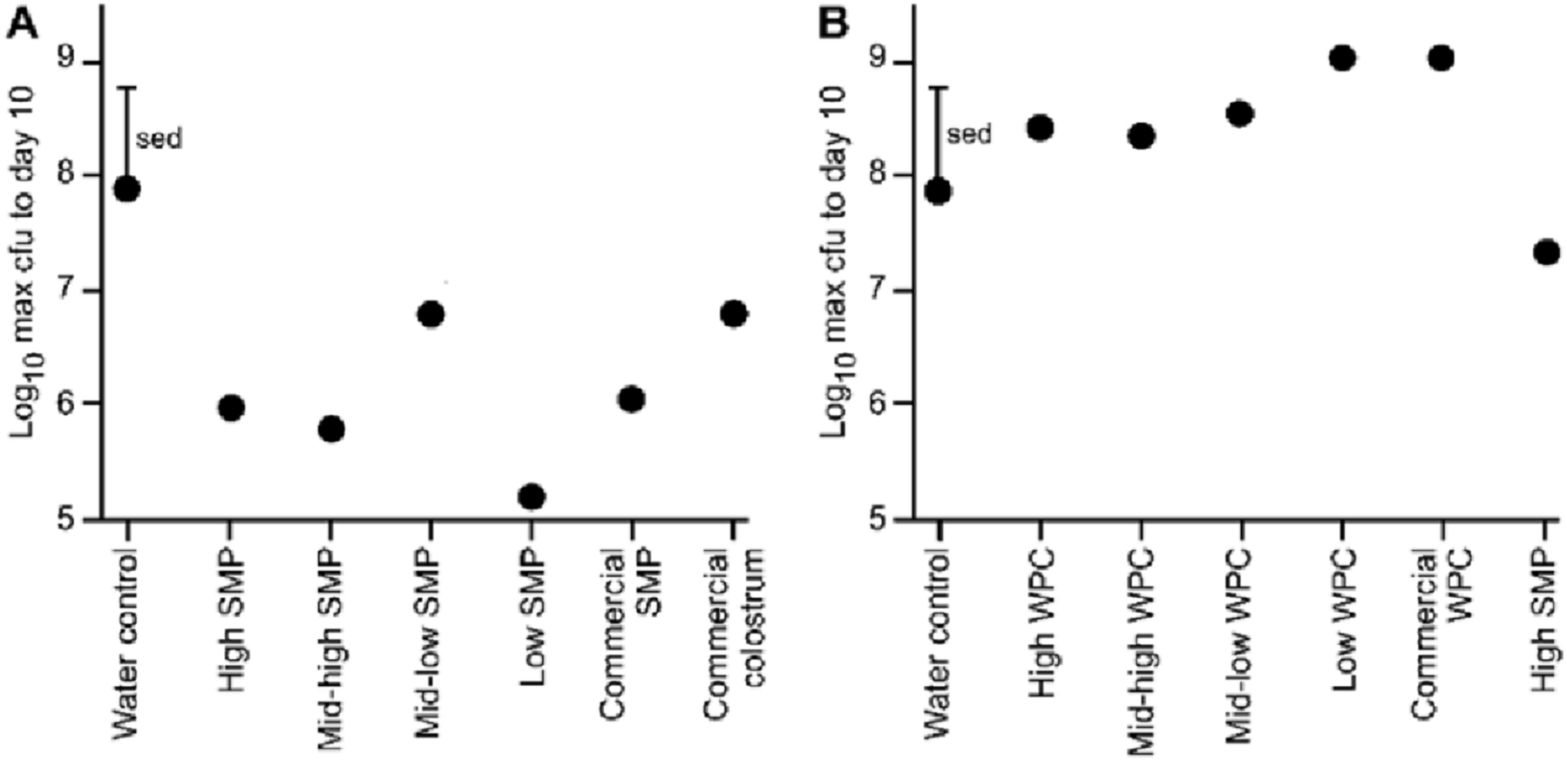
Comparison of maximum bacterial viable counts between mice supplemented with SMP or WPC. *C. rodentium* was enumerated from faecal collections (cfu/g faeces). (A) Peak *C. rodentium* shedding in mice supplemented with treatments of SMP within the 10 day experimental period. The mean viable count for each treatment and a standard error of treatment difference (sed) is presented. *n* = 16 per treatment group. (B) Peak *C. rodentium* shedding in mice supplemented with treatments of WPC within the 10 day experimental period. The mean viable count for each treatment and a standard error of treatment difference (sed) is presented. *n* = 16 per treatment group.

All animals in each trial received the *C. rodentium* within 1 h of each other, however, maximum excretion did not occur on the same day, either within groups, or between groups. Therefore, we compared the maximum *C. rodentium* viable count from faecal pellets for each of the groups up to day 10 (enabling direct comparison of the two trials). These data illustrate a reduction in bacterial shedding in the groups given the SMP preparation compared to water (Fig. 2A). Conversely in the WPC trial, *C. rodentium* enumerated from the WPC treatments were not significantly different from the infection water-fed control (Fig. 2B; Table 3). However, the High SMP group in the WPC trial had reduced bacterial shedding, approaching statistical significance (ANOVA *p* = 0.01, Fishers ULSD, Water (infection), ab; High, mid-high, mid-low, bc; low, c; SMP, a; commercial WPC, c, where mean values with unlike letters are significantly different (*p* < 0.05)). The SMP treatment was significantly different (less *C. rodentium* shedding) compared to other treatment groups.

**Table 3:** Summary of infection rates and magnitude.

Treatment	Number of mice		Viable count (mean log cfu/g faeces)		Viable count (log max cfu/g faeces up to day 10)	
SMP	Non-productive infection	Infected	Day 10	SEM	Day 10	SEM
Water		16	7.86	0.56	7.89	0.55
SMP high*	3	13	5.72	0.66	5.97	0.64
SMP mid-high	2	14	5.26	0.74	5.77	0.68
SMP mid-low	1	15	6.12	0.74	6.79	0.63
SMP low	6	10	4.85	0.65	5.18	0.69
Colostrum	2	14	5.73	0.55	6.80	0.52
SMP commercial		16	5.32	0.65	6.05	0.63
Water no infection	8		0	0	0	0
ANOVA			sed 0.92, *p* = 0.04		sed 0.88, *p* = 0.07	
**WPC**						
Water		16	7.37	0.57	7.87	0.44
WPC high		16	7.89	0.43	8.42	0.41
WPC mid-high		16	7.74	0.38	8.37	0.39
WPC mid-low		16	8.09	0.29	8.56	0.33
WPC low		16	8.60	0.20	9.05	0.15
SMP high*		16	6.86	0.61	7.32	0.56
WPC commercial		16	8.74	0.15	9.1	0.09
Water no infection	8		0	0	0	0
ANOVA			sed 0.58, *p* = 0.02		sed 0.52, *p* = 0.01	

**Notes:**

Blend ratios (%) of treatments (high:low) are described in Table 1. Non-productive infection indicates that Citrobacter was detected but at low cfu, and with no increase over time.

SMP, skimmed milk powder; WPC, whey protein concentrate; Ig immunoglobulin.

* Indicates the same product; sed, standard error of the difference between means of groups.

## Discussion

Milk components are often studied individually in their pure form, yet their combined or additive activities within the milk matrix are less well understood. For this study we chose IgA, IgG and Lf as selected markers of milk bioactive content, because they are found in all mammalian milk, and are important for GI and immune development and function (Blewett et al., 2008; Korhonen, Marnila & Gill, 2000). It could be reasoned that bovine proteins are not effective in a non-bovine system, but in recent studies purified bovine sIgA and purified human sIgA have been shown to have equal capacity to bind both pathogenic and non-pathogenic bacteria, suggesting this is unlikely to be the case (Hodgkinson et al., 2017).

Our novel study explored two different functionalities: the milk products which differed by selected bioactive protein concentration, and the format of the different milk matrices: that of SMP, and WPC (Table 1).

We tested the influence of the respective products on *C. rodentium* shedding, using the well-established model of *C. rodentium* in mice (Collins et al., 2014). Our results suggest SMP but not WPC may ameliorate progression of *C. rodentium* faecal shedding, indeed the WPC appeared to increase bacterial shedding. This could indicate exacerbation of infection since cfu titre exceeded that seen in the SMP trial despite inoculation with the same dose of *C. rodentium*. The ‘water-fed infection control groups’ were similar in both studies.

We based our experimental design on the work of others which used faecal counts as a measure of infection (Wiles, Dougan & Frankel, 2005). However, although the increase of faecal excretion, and the time for eradication is related to anti-infectious traits, this is not conclusive evidence of infection. We observed that components within SMP could interrupt the infection kinetics of *C. rodentium* whilst WPC did not. It is possible that WPC could be advantageous to the growth of *C. rodentium* and this is an interesting area for future study (Bury, Jelen & Kimura, 1998; Pineda-Quiroga et al., 2018). The potential of products to enhance or diminish bacterial success is highly relevant to the growing personalised nutrition market, and for the medical researchers seeking alternatives to antibiotics, for example. In the SMP trial, we followed the infection for 10 days, however, the infection was not resolved in this timeframe. In the WMP trial, we extended our observations for an extra four days. Although the infection appeared to have reached a peak by Day 10, it was still not resolved by Day 14. These data allowed us to compare treatment effects on the establishment of the infection but not the resolution or clearance of the infection. Following the infection out to resolution would also provide additional important information on the effect of both matrix and selected bioactives on the infection kinetics. However, it would not have changed our conclusions about the treatment effect on infection establishment and progression. Further work is required to understand mechanisms of action.

We had hypothesized that the increased abundance of the whey proteins (represented by IgA, IgG and Lf) in WPC compared to SMP would result in an increased efficacy of the product, as measured by reduction in *C. rodentium* shedding. Our results provided no evidence for this. Surprisingly, we failed to observe any beneficial effect of WPC on *C. rodentium* shedding in the animal model; indeed some of the WPC treatments were associated with a higher level of bacterial shedding than water alone. This was despite increased levels of IgA (fourfold), IgG (threefold) and Lf (over threefold). This suggests that formulation could affect protein functionality, and that simply increasing the selected bioactive levels in a product does not necessarily improve functionality. Both SMP and WPC trials was designed to demonstrate the extent that the selected bioactive molecules (IgA IgG and LF) affect *C. rodentium* infection by titration of the high IgA (IgG and LF) products with a low IgA, (IgG and LF) counterpart product. In vitro ELISA assays revealed binding activity against *C. rodentium* of IgA, IgG and Lf antibodies in the high WPC milk products, commercial WPC and also the high SMP products (Fig. S5). However, that we found no difference between doses would indicate that the effects we observed were not due to the selected bioactive components in the treatments. This in turn suggests that the effect was due to an unidentified component that was constant in all the SMP treatments and we termed this the ‘milk effect’.

The protective effect of SMP may originate from the proteins that are removed in the course of WPC manufacture. Up to 80% of the protein content of liquid milk and reconstituted SMP is casein (αs1-, αs2-, β-, and κ-casein). The caseins and the minerals in milk form structures called micelles. These structures can act as chaperones that help stabilise proteins, such as β-lactoglobulin (Holt et al., 2013). This protects the proteins from the effects of heat or pH change (Morgan et al., 2005) and helps to prevent aggregation and precipitation of other proteins found in the whey (Bhattacharyya & Das, 1999) so retaining the bioactivity of the protein. Precipitation of casein by enzyme (Barth & Behnke, 1997) or acid (Walstra & Jenness, 1984) leaves behind the whey fraction containing soluble proteins (15–20%) from which WPC is produced. It is possible that the protective ‘milk effect’ seen with SMP had been negated in WPC through the deletion of key protective components during the manufacturing process, either by removal or inactivation. Removal of casein during WPC manufacture, along with minerals and lactose, may make the proteins more vulnerable to enzyme activity, heat and/or pH change. For example, it has been demonstrated that digestion of milk is affected by the way in which it is processed (Tunick et al., 2016).

Whey protein concentrate is widely used in health and sports supplements and is recognised as a nutritious and healthy protein source (Patel, 2015). It is easily digested and has a high biological value (Hoffmann & Falvo, 2004). The apparent negative effect of the WPC treatment in this animal model is intriguing. WPC is known to aid growth and viability of certain fermentative bacteria (Bury, Jelen & Kimura, 1998; Zisu & Shah, 2003) by providing an additional nitrogen source. However, the direct effects of WPC on bacteria such as *C. rodentium* have not been documented to our knowledge.

In order to be successful *C. rodentium* needs to adhere to the GI tract where upon it adapts to the GI environment and undergoes a virulence switch to facilitate colonization of the distal colon and rectum (Wiles et al., 2004, 2006). In a recent study it was demonstrated that gut motility was reduced in rats fed WPC (Dalziel et al., 2016). We suggest that this could, in part, explain the apparent success of *C. rodentium* in the WPC fed mice, where reduced gut motility may have favoured pathogen colonisation. Future studies need to test this hypothesis.

There were no technical issues with the gavaging and all animals received the same inoculum, yet we saw variation in faecal shedding within groups. In the SMP experiment there was a number of mice that were not infected, despite administration of a dose of *C. rodentium* (2.0E+09 cfu), which should be sufficient to give a uniform infection. This same effect was not observed in the WPC trial which raises the possibility that failure to infect was due to the presence of SMP matrix. Mice were housed in cages up to four individuals, so this phenomenon may also reflect differences in an individual animal’s grooming habits or levels of coprophagia. Similarly it could reflect the individuals’ susceptibility to infection, despite being from the same genetic background. It is also possible that ingestion of *C. rodentium* shed in faeces could have increased virulence after passing through the gut (Wiles, Dougan & Frankel, 2005) (Table 3).

For SMP fed mice, differences were observed between the treatment groups on days 8 and 10 (Fig. 1A). The mean levels of excreted *C. rodentium* for the each of the SMP groups was lower than water-fed infected controls and this was significant for all doses, except for the mid-low treatment. This indicates that SMP had a suppressive effect on bacterial shedding (an SMP ‘milk effect’), reducing the level of bacterial shed from the mouse intestine but with no significant selected bioactive dose effect. This may have been as a result of the highest dose being insufficient to see a different effect, or that increased bioactive content is not beneficial in this model.

## Conclusion

Together these data highlight the important influence of the food matrix and that how food is manufactured may influence the health effects of foods. The study set out to investigate the contribution of selected bioactive levels (IgA, IgG and Lf) in SMP and WPC on *C. rodentium* shedding, in a model of GI infection. However, the dominant finding was the profound ‘milk effect’ observed with SMP and that the SMP and WPC products had contrasting effects. Further studies will elucidate the pathways that lead to this observation.

## Supplemental Information

10.7717/peerj.5359/supp-1 Supplemental Information 1 Supplementary information. **Figure S1. Establishment of *C. rodentium* infection in the mouse GI tract**.
*Fecal pellets were collected from mice infected with C. rodentium (2 × 10^9^ cfu) introduced via oral gavage. The average (± standard errors) number of cfu of nalidixic acid-resistent bacteria present per gram of faecal pellets from inoculated mice (n = 4) over time are presented*.
**Figure S2. Bioactive levels of SMP, WPC and commercial products after reconstitution**.
*SMP and WPC were prepared as described in the main text*.
*SMP High and Low products were manufactured from the milk of cows with naturally higher or lower levels of the bioactive molecules IgA, IgG and Lf. The low bioactive SMP was used as a diluent to titrate the selected bioactive levels in the high SMP*.
*WPC High product was manufactured from the milk of cows with naturally higher levels of the bioactive molecules IgA, IgG and Lf. Low WPC was heat treated commercial product. High and Low WPC were blended to produce a range of bioactive levels*.
*Milk treatments were normalised to 3.2% protein*.
**Figure S3. Average weight of mice (g) by group challenged with *Citrobacter rodentium* and treated with SMP**.
*Following gavage (infection) mice were weighed daily for 4 days and then every second day until end of trial. Day 0 depicts infection day and + denotes infection. Mean weights are adjusted for initial weight at day 0. The standard error of treatment difference (sed) is presented. N = 16 for infection/treatment groups (+), n = 8 for water no infection control (grey line)*.
**Figure S4. Average weight of mice (g) by group, challenged with *C. rodentium* and treated with WPC**.
*Following gavage (infection) mice were weighed daily for 4 days and then every second day until end of trial. Day 0 depicts infection day and + denotes infection. Mean weights are adjusted for initial weight at day 0. The standard error of treatment difference (sed) is presented. N = 16 for infection/treatment groups (+), n = 8 for water no infection control (grey line).*.
**Figure S5. *C. rodentium-specific antibody titres detected in milk products***.
*Microtitre plates were incubated overnight at 4–8 °C with 100μl/well of heat killed C. rodentium (5 × 10^8^/mL). Plates were blocked with PBS-T, 1% (w/v) bovine serum albumin and serial dilutions of the milk treatments added at 1:100, 1:1000, 1:10 000 and 1:100 000; 100 μl. Sheep anti-bovine IgA, IgG and goat anti-bovine Lactoferrin conjugated with horseradish peroxidase (Bethyl laboratories) were added at 1:1000, 1:5000 and 1:10 000 dilutions, respectively. TMB One Component HRP Microwell substrate solution was used (BioFX Laboratories, 100 μl) and the reaction stopped with 50 μl of 2M-H2SO4. Optical density was measured at 450nm using an automated plate reader (Versa-Max; Molecular Devices). Results are expressed as absorbance at 450nm.*.
**Table S1. SMP treatment effect on *C. rodentium* (cfu)**.
*SMP trial, mean log cfu by treatment for each day. Analysis of variance (p<0.05), and Fisher’s ULSD test; mean values with unlike letters are significantly different (p<0.05)*.
**Table S2. SMP treatment effect on maximum *C. rodentium* (cfu) by day 10**.
*SMP trial, mean log Max cfu by day 10. Analysis of variance (p<0.05), and Fisher’s ULSD test; mean values with unlike letters are significantly different (p<0.05)*.
**Table S3. WPC treatment effect on *C. rodentium* (cfu)**.
*WPC trial mean log cfu by treatment for each day. Analysis of variance (p<0.05), and Fishers ULSD test; mean values with unlike letters are significantly different (p<0.05)*.
**Table S4. WPC treatment effect on maximum *C. rodentium* (cfu) by day 10**.
*WPC trial mean log max cfu by day 10. Analysis of variance (p<0.05), and Fisher’s ULSD test; mean values with unlike letters are significantly different (p<0.05)*.

10.7717/peerj.5359/supp-2 Supplemental Information 2 Raw data SMP cfu. Faecal pellets were collected every second day, and homogenised to achieve a suspension of 100 mg pellet per ml. A series of dilutions of the suspension were plated onto LB-agar containing nalidixic acid. The colonies (viable count) present after incubation at 37 °C for 24 h were enumerated.

10.7717/peerj.5359/supp-3 Supplemental Information 3 Raw data WPC cfu. Faecal pellets were collected every second day, and homogenised to achieve a suspension of 100 mg pellet per ml. A series of dilutions of the suspension were plated onto LB-agar containing nalidixic acid. The colonies (viable count) present after incubation at 37 °C for 24 h were enumerated.

## Additional Information and Declarations

### Data Availability

The following information was supplied regarding data availability: The raw data are provided in the Supplemental Files.
