# Supplementary material for: Bovine milk derived skimmed milk powder and whey protein concentrate modulates Citrobacter rodentium shedding in the mouse intestinal tract
Source: PeerJ. 2018 Jul 27;6:e5359. doi: 10.7717/peerj.5359 (PMC6065463; doi:10.7717/peerj.5359)
Supplement: Supplemental Information 1 — Figure S1. Establishment of C. rodentium infection in the mouse GI tract. Fecal pellets were collected from mice infected with C. rodentium (2 × 10^9^ cfu) introduced via oral gavage. The average (± standard errors) number of cfu of nalidixic acid-resistent bacteria present per gram of faecal pellets from inoculated mice (n = 4) over time are presented. Figure S2. Bioactive levels of SMP, WPC and commercial products after reconstitution. SMP and WPC were prepared as described in the main text. SMP High and Low products were manufactured from the milk of cows with naturally higher or lower levels of the bioactive molecules IgA, IgG and Lf. The low bioactive SMP was used as a diluent to titrate the selected bioactive levels in the high SMP. WPC High product was manufactured from the milk of cows with naturally higher levels of the bioactive molecules IgA, IgG and Lf. Low WPC was heat treated commercial product. High and Low WPC were blended to produce a range of bioactive levels. Milk treatments were normalised to 3.2% protein. Figure S3. Average weight of mice (g) by group challenged with Citrobacter rodentium and treated with SMP. Following gavage (infection) mice were weighed daily for 4 days and then every second day until end of trial. Day 0 depicts infection day and + denotes infection. Mean weights are adjusted for initial weight at day 0. The standard error of treatment difference (sed) is presented. N = 16 for infection/treatment groups (+), n = 8 for water no infection control (grey line). Figure S4. Average weight of mice (g) by group, challenged with C. rodentium and treated with WPC. Following gavage (infection) mice were weighed daily for 4 days and then every second day until end of trial. Day 0 depicts infection day and + denotes infection. Mean weights are adjusted for initial weight at day 0. The standard error of treatment difference (sed) is presented. N = 16 for infection/treatment groups (+), n = 8 for water no infection control (grey line).. Figur [file peerj-06-5359-s001.pdf]

Supplementary data

Bovine milk derived skimmed milk powder and whey protein concentrate modulates infection by *Citrobacter rodentium* in the mouse intestinal tract.

Running title: SMP, WPC and bacterial infection

Cakebread, J. A[1],* Hodgkinson, A. J[1], Wallace, O[1], Callaghan, M[1], Hurford, D[1], Wieliczko, R[1], Harris, P[1] and Haigh, B[1,2]

1. Dairy Foods Team, Food and Biobased products, AgResearch, Hamilton, New Zealand

2. Miraka, Taupo, New Zealand

Tel: +64 7 838 5317

Fax:+64 7 838 5012

PeerJ

Address correspondence to:

*Julie A Cakebread, Julie.cakebread@agresearch.co.nz

Supplementary data

Figure S1.Establishment of *C. rodentium* infection in the mouse GI tract.

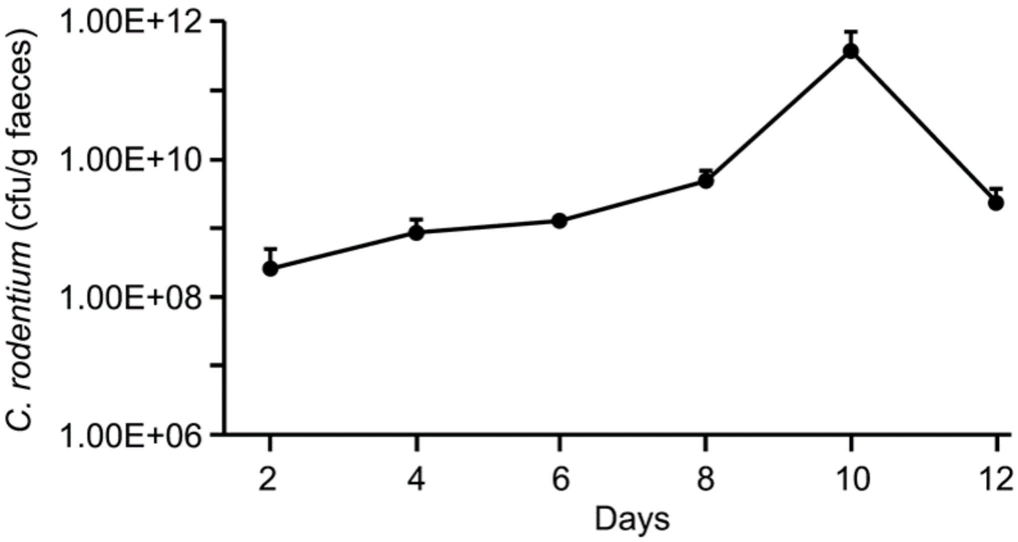

Supplementary data

Figure S2 Bioactive levels of SMP, WPC and commercial products after reconstitution

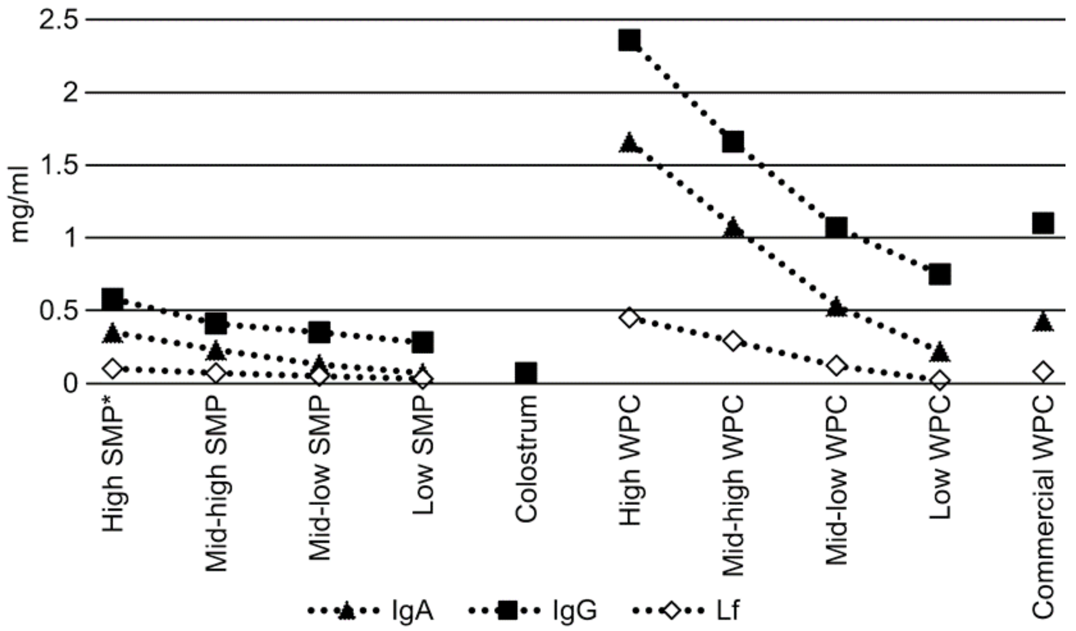

Supplementary data

Figure S3 Average weight of mice (g) by group challenged with *Citrobacter rodentium* and treated with SMP

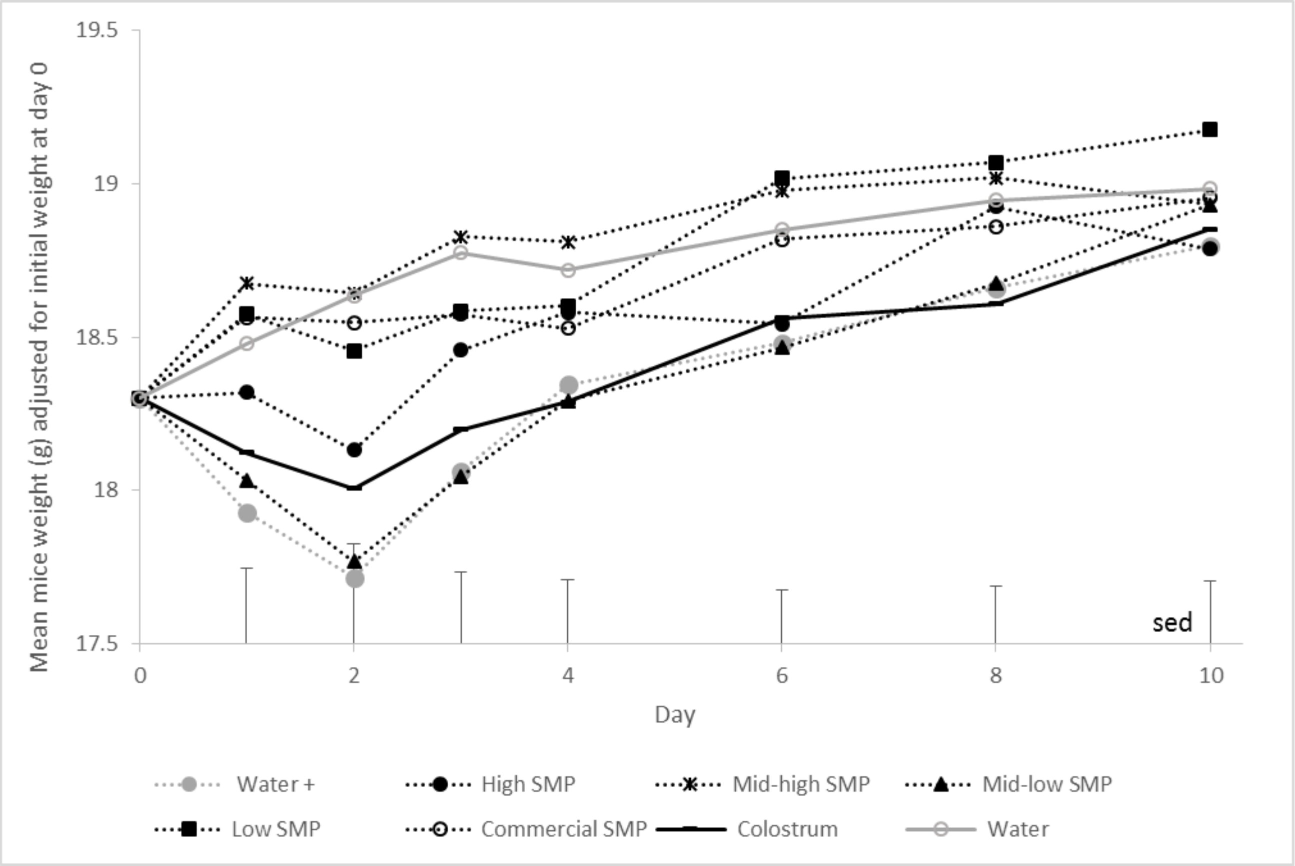

Supplementary data

Figure S4 Average weight of mice (g) by group, challenged with *C. rodentium* and treated with WPC

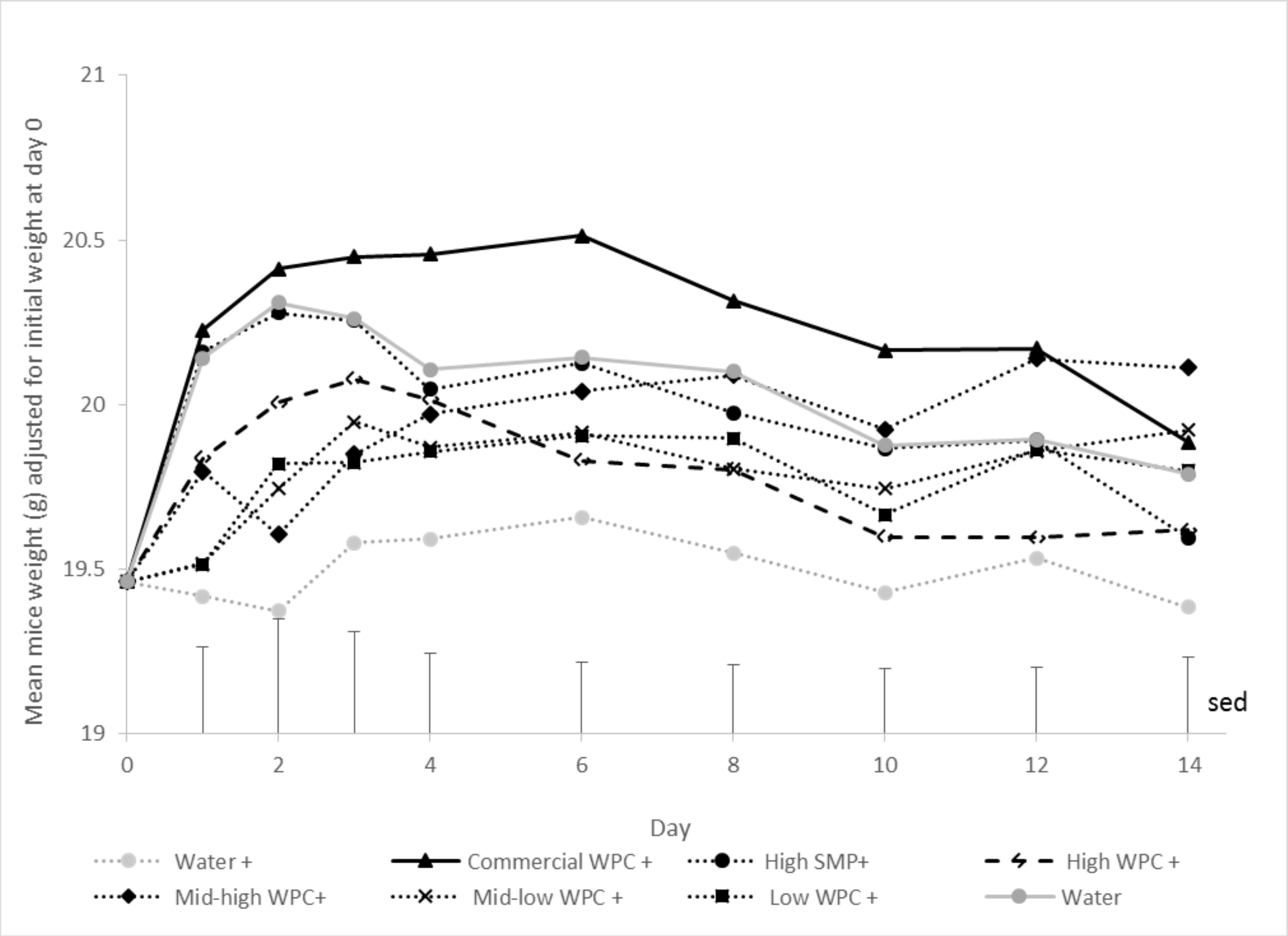

Supplementary data

Figure S5 *C. rodentium -specific antibody titres detected in milk products*

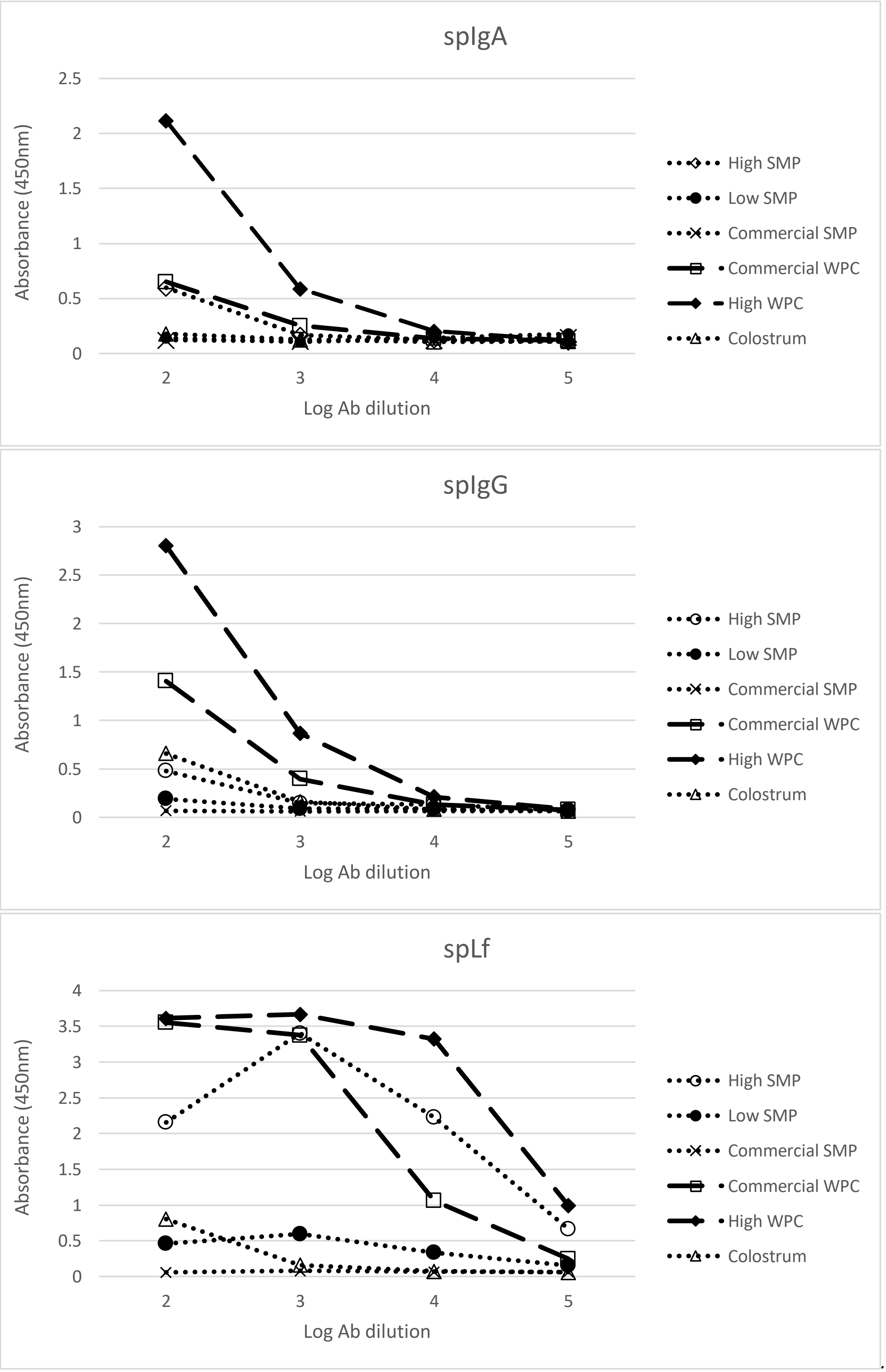

Supplementary data

Table S1 SMP treatment effect on *C. rodentium* (cfu)

SMP	Day 2		Day 4		Day 6		Day 8		Day 10	
ANOVA		p=0.70		P=0.16		P=0.15		P=0.04		P=0.04
Treatment	Mean log cfu	Fisher's ULSD	Mean	Fisher's ULSD	Mean	Fisher's ULSD	Mean	Fisher's ULSD	Mean	Fisher's ULSD
Water	4.5	a	6.04	b	7.0	b	7.5	c	7.9	b
SMP high*	4.0	a	4.2	a	5.1	a	5.4	ab	5.7	a
SMP mid-high	4.1	a	4.7	ab	5.3	ab	5.2	ab	5.3	a
SMP mid-low	4.6	a	4.8	ab	5.7	ab	6.1	abc	6.1	ab
SMP low	3. 8	a	3.9	a	4.3	a	4.4	a	4.9	a
Colostrum commercial	4.3	a	5.0	ab	5.7	ab	6.4	bc	5.7	a
SMP Commercial	4.0	a	4.6	ab	5.0	a	5.3	ab	5.3	a

Table S2 SMP treatment effect on maximum *C. rodentium* (cfu) by day 10

**log max cfu**	ANOVA	0.068
**Treatment**	**Mean**	**Fisher’s ULSD**
Water	7.893	b
SMP High	5.966	a
SMP mid-high	5.767	a
SMP mid-low	6.791	ab
SMP low	5.176	a
Colostrum commercial	6.802	ab
SMP Commercial	6.045	a

Supplementary data

Table S3 WPC treatment effect on *C. rodentium* (cfu)

**WPC**	**Day 2**		**Day 4**		**Day 6**		**Day 8**		**Day 10**		**Day 12**		**Day 14**	
**ANOVA**		p=0.18		P=0.007		P=0.002		P=0.02		P=0.02		P=0.03		P=0.13
**Treatment**	**Mean log cfu**	**Fisher's ULSD**	**Mean**	**Fisher's ULSD**	**Mean**	**Fisher's ULSD**	**Mean**	**Fisher's ULSD**	**Mean**	**Fisher's ULSD**	**Mean**	**Fisher's ULSD**	**Mean**	**Fisher's ULSD**
Water	4.6	ab	5.7	a	6.7	ab	7.2	ab	7.4	ab	7.9	b	7.7	ab
WPC high	5.4	b	7.1	bc	8.2	cd	7.8	abc	7.9	abc	7.6	ab	7.3	a
WPC Mid-high	5.3	ab	6.6	ab	7.2	abc	8.1	bc	7.7	abc	8.0	b	7.7	ab
WPC Mid-low	5.4	b	6.6	abc	7.6	bcd	8.3	bc	8.1	bc	8.1	b	7.8	ab
WPC low	5.4	ab	7.3	bc	8.0	cd	8.5	bc	8.6	c	8.4	b	8.1	ab
SMP high*	4.3	a	5.7	a	6.3	a	6.6	a	6.6	a	6.8	a	7.2	a
WPC Commercial	5.6	b	7.9	c	8.5	d	8.9	c	8.7	c	8.6	b	8.4	b

Table S4 WPC treatment effect on maximum *C. rodentium* (cfu) by day 10

**Day 10 log max cfu**	ANOVA	p=0.01
**Treatment**	**Mean**	**Fisher's ULSD**
Water	7.9	ab
WPC high	8.4	bc
WPC Mid-high	8.4	bc
WPC Mid-low	8.6	bc
WPC low	9.0	c
SMP high*	7.3	a
WPC Commercial	9.1	c
